# Dynamin2 mutations in newly diagnosed acute myeloid leukemia: clinical characteristics, and prognostic significance

**DOI:** 10.1186/s40164-025-00628-5

**Published:** 2025-03-21

**Authors:** Kunpeng Luo, Jiayuan Chen, Wenting Wang, Yan Hui, Shaowei Qiu, Bingcheng Liu, Yingchang Mi, Jianxiang Wang, Hui Wei

**Affiliations:** 1 State Key Laboratory of Experimental Hematology, National Clinical Research Center for Blood Diseases, Haihe Laboratory of Cell Ecosystem, Institute of Hematology & Blood Diseases Hospital, Chinese Academy of Medical Sciences & Peking Union Medical College, Nanjing Rd 288, Tianjin, 300020 China; 2 Tianjin Institutes of Health Science, Tianjin, 301600 China

**Keywords:** Acute myeloid leukemia, Gene mutation, *DNM2*, Next generation sequencing, Clinic outcome, *CEBPA*, *RUNX1::RUNX1T1*

## Abstract

Acute myeloid leukemia (AML) is a highly heterogeneous myeloid malignancy which can be classified by genetic aberrations. To evaluate the impact of the dynamin 2 mutation in AML, we systematically assessed the characteristics and prognostic of *DNM2* mutated patients in AML. In 912 AML patients, 20 somatic mutations in the *DNM2* gene were identified among the 18 *DNM2* mutated AML patients (2%). Of the mutation events, 60% (12/20) were in the dynamin central region of *DNM2*. *DNM2*mutations were preferentially occurred in AML with *CEBPA* mutation (11/18, 61.1%), or *RUNX1::RUNX1T1* fusion gene (6/18, 33.3%). *DNM2* mutations were associated with better overall survival (*P* = 0.028), event-free survival (*P* = 0.0093) and trends towards better relapse-free survival (*P* = 0.08), which seems potentially attribute to its coexisting with *CEBPA* mutation and *RUNX1::RUNX1T1* fusion gene. Our study demonstrated the clinical characteristics and the role of *DNM2* mutations in AML, which might facilitate understanding the pathogenesis of AML.

**Supplementary Information:**

The online version contains supplementary material available at 10.1186/s40164-025-00628-5.

## To the editor,

Acute myeloid leukemia (AML) is a highly heterogeneous myeloid malignancy, which can be classified by genetic aberrations [1–5]. The exploration of novel prognostic indicators and the elucidation of co-occurrence patterns can provide valuable support for the clinical precision treatment of AML [6–8]. Dynamin 2 (*DNM2*) is a major member of the large GTPase superfamily, which consists of four major functional domains: the Ras-like GTPase, Dynamin central region, PH domain, and GTPase effector domain. It is ubiquitously expressed and plays a pivotal role in membrane remodeling processes, including endocytosis, intracellular vesicle trafficking, and exocytosis [9, 10]. *DNM2* mutations plays a role in multiple types of cancer. Emerging research has revealed its regulatory function in AML [9, 11]. However, the genetic landscape of *DNM2* mutations in AML has not been fully characterized. In this study, we investigated the characteristics and prognostic value of *DNM2* mutation in AML.

**Fig. 1:**
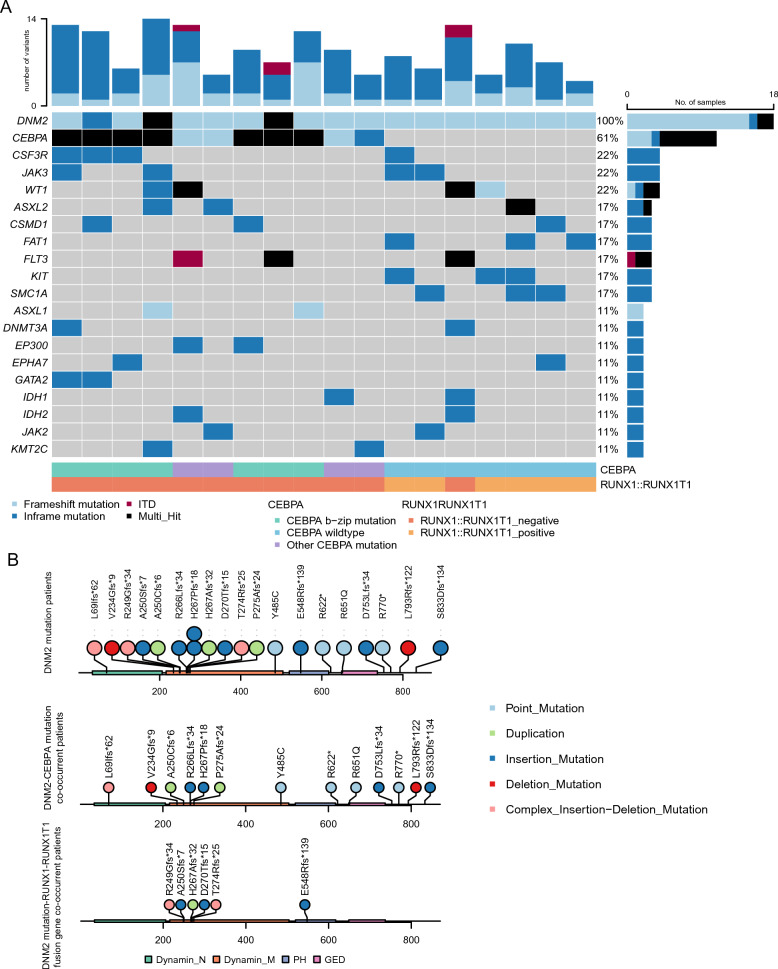
Mutational status of AML patients with *DNM2* mutation. Mutational landscape of AML patients with *DNM2* mutation (**A**). Each column represents a patient; each colored box indicates a specified somatic mutation. Light gray represents the wild-type cases. Bar plots indicate the mutation frequency of relevant gene. Bottom exhibited the *RUNX1::RUNX1T1* fusion gene status and *CEBPA* mutation status of AML patients. *DNM2* mutations performs different localization pattern in AML patients with *CEBPA* mutation and *RUNX1::RUNX1T1* fusion gene (**B**). Domain structure and *DNM2* mutation sites in AML patients with *DNM2* mutations, AML patients with *DNM2* and *CEBPA* mutations, and AML patients with *DNM2* mutations and *RUNX1::RUNX1T1* fusion gene. Dynamin_N, Ras-like GTPase domain; Dynamin_M, Dynamin central region; PH, PH domain; GED, GTPase effector domain

Out of a total of 1003 non-APL AML patients who underwent intensive chemotherapy, 33 patients lacking NGS data and 58 patients who were not newly diagnosed with AML were excluded. Consequently, 912 patients were included in the analysis (Figure S1). Of 912 patients, 18 are *DNM2* mutation and 894 are *DNM2* wildtype patients (Table S1 and Figure S1). No significant differences in baseline characteristics between the *DNM2* mutant and wild-type groups were found (Table S1). Compared to patients with wildtype *DNM2,* those with *DNM2* mutations had a higher frequency of *CEBPA* mutations (*P* < 0.001, Table S1). We illustrated the mutational spectrum in patients with *DNM2* mutations (Fig. 1A and S2). Among the identified mutations, *CEBPA* had the highest co-occurrence rate at 61.1% (11/18), followed by *RUNX1::RUNX1T1* fusion gene (n = 6, 33.3%), *CSF3R* (n = 4, 22.2%), *JAK3* (n = 4, 22.2%) and *WT1* (n = 4, 22.2%), which suggested that *DNM2* mutations occurred preferentially in AML with *CEBPA* mutation, or *RUNX1::RUNX1T1* fusion gene. *RUNX1::RUNX1T1* fusion gene and *CEBPA* mutations are mutually exclusive in *DNM2* mutated patients. In patients with *DNM2* and *CEBPA* mutations, 63.6% (7/11) harbored *CEBPA* b-ZIP mutation, 36.4% (4/11) had other types of *CEBPA* mutation. Totally, 20 somatic mutations in the *DNM2* gene were identified among the 18 *DNM2* mutated AML patients (Fig. 1B). Of the mutation events, 60% (12/20) were in the dynamin central region. The Ras-like GTPase domain, the PH domain, and the Ras-like GTPase effector domain each had one mutation event (5%). Given that most of DNM2 mutations occur in the dynamin central region, which plays a key role in membrane remodeling and vesicle formation, these mutations may affect critical cellular processes in AML and result in the clinic significance of DNM2 mutation. In patients with DNM2 mutation and RUNX1::RUNX1T1 fusion gene, 83.3% (5/6) of mutations were preferentially located in the dynamin central region, with the rest in the PH domain.

**Fig. 2:**
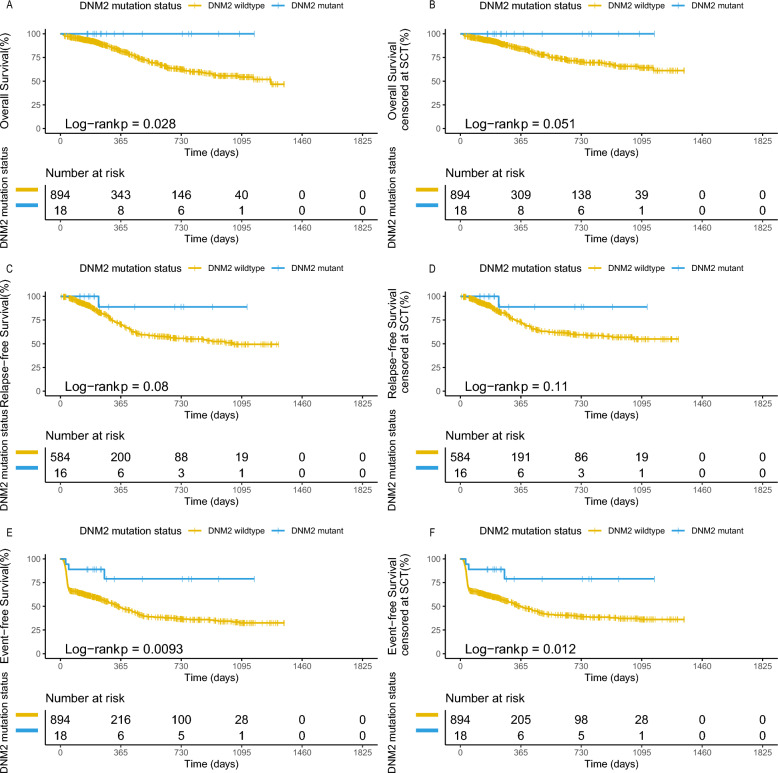
Prognostic significance of *DNM2* mutation in AML patients. Comparison of OS (**A**), RFS (**C**) and EFS (**E**) between *DNM2* mutated and wild-type AML patients. Comparison of OS (**B**), RFS (**D**) and EFS (**F**) censored at the time of transplantation between *DNM2* mutated and wild-type AML patients

We then analyzed the prognostic effects of *DNM2* mutations in AML patients (Fig. 2). *DNM2* mutated patients demonstrated better OS (*P* = 0.028) and EFS (*P* = 0.0093) and trends towards better RFS (*P* = 0.08) (Fig. 2A, C and E). Similar results were found when censored at the time of transplantation (Fig. 2B, D and F). However, no statistical significance was observed in the case–control matching analysis (Figure S3). Then, we tested prognostic significance of *DNM2* mutation in different ELN risk subgroups (Figure S4 and S5). We did not analyze the effect in ELN adverse group, since there are only two patients with *DNM2* mutation in adverse group. *DNM2* mutation did not significantly affect outcomes in ELN favorable and intermediate risk group, although *DNM2* mutant was associated with better EFS in ELN intermediate groups (Figure S4 and S5). These results indicate that the prognostic significance of the *DNM2* mutation may stem from its coexistence with the *CEBPA* mutation and *RUNX1::RUNX1T1* fusion gene. We further explored the prognostic significance of *DNM2* mutation in *CEBPA* mutation and *RUNX1::RUNX1T1* fusion gene subgroups (Figure S6 and S7). In subgroup of *CEBPA* mutation, patients with *DNM2* mutation showed better EFS, but not RFS or OS, compared to those with *DNM2* wild-type (Figure S6). *DNM2* mutation didn’t affect prognosis in patients with the *RUNX1::RUNX1T1* fusion gene (Figure S7). Finally, we conducted the multivariate Cox regression analysis (Figure S8). Due to the absence of events in DNM2-mutated patients in the OS analysis, only EFS and RFS were included in the multivariate analysis.DN*M2* mutation correlated with better EFS (*P* = 0.02, Figure S8A) but not RFS (Figure S8B) in multivariate analysis.

The study still has some limitations. While the sample size for DNM2 mutation cases is small, restricts the robustness of our findings, it is needed to validate these findings through multi-center studies in the future. In this study, we found that the majority of the *DNM2* mutations occurred in the dynamin central region in AML patients. Patients with *DNM2* mutation demonstrated a strong preference for coexisting with *RUNX1::RUNX1T1* fusion gene and *CEBPA* mutation. *DNM2*’s mutations correlated with better outcomes, which may attribute to this coexisting pattern. Our results demonstrated the clinical characteristics of patients with *DNM2* mutations, which might facilitate understanding the pathogenesis of AML.

## Supplementary Information

Additional file 1.

Additional file 2.

Additional file 3.

Additional file 4.

Additional file 5.

Additional file 6.

Additional file 7.

Additional file 8.

Additional file 9.

Additional file 10.

## Data Availability

No datasets were generated or analysed during the current study.
