# Supplementary material for: Dynamin2 mutations in newly diagnosed acute myeloid leukemia: clinical characteristics, and prognostic significance
Source: Exp Hematol Oncol. 2025 Mar 21;14:42. doi: 10.1186/s40164-025-00628-5 (PMC11927327; doi:10.1186/s40164-025-00628-5)
Supplement: Supplementary file 1 — Additional file 1. [file 40164_2025_628_MOESM1_ESM.docx]

**Method**

**Patients**

In total, of the 1003 non-APL consecutive patients who received intense chemotherapy, 912 with newly diagnosed AML were included in this study. Patients were treated in Institute of Hematology & Blood Diseases Hospital, Chinese Academy of Medical Sciences & Peking Union Medical College between 2019/1/10 and 2024/1/11. All patients received intensive induction therapy with the combination of cytarabine and anthracycline ± homoharringtonine or venetoclax, or cytarabine plus aclacinomycin ± G-CSF, followed by 3-4 courses of consolidation regimens with high, intermediate or standard dose cytarabine after complete remission. Intrathecal injections were administered for routine prophylaxis against central nervous system leukemia. Clinic information and related laboratory data were obtained from the medical records. The study was approved by institutional ethics committee (Blood Diseases Hospital Ethics Committee). The overall design and conduction of the research was accordance with the Declaration of Helsinki. Written informed consent was obtained from all enrolled participants.

**Next-generation sequencing (NGS)**

Genomic DNA was extracted and subjected to polymerase chain reaction (PCR) amplification. The amplified DNA was analyzed for mutations using next-generation sequencing (NGS). The DNM2 gene was evaluated in all samples, with comprehensive coverage of the entire coding region, achieved through customized liquid-phase probes designed for targeted capture of the full coding sequence (CDS). NGS was performed for all patients, with a median sequencing depth of over 2000x to ensure high sensitivity and accuracy in mutation identification. The average gene coverage of 98% was calculated by first determining the average sequencing depth and then measuring the proportion of the target capture region covered by at least 0.2x depth.

**Variant calling procedure**

The quality control of the FASTQ data was carried out using Fastp (version 0.23.2, <https://github.com/OpenGene/fastp>), which removed terminal adaptor sequences and low-quality reads from the raw data. Data alignment and mutation calling were performed using Dragen (version 3.10.4, Illumina), a local installation hardware-accelerated sequencing processing pipeline. Somatic mutations were identified based on a VAF exceeding 0.5%, and an in-house algorithm was used to review hotspot variants. The final candidate variants were manually verified using Integrative Genomics Viewer (IGV, <https://www.igv.org/>). Copy-number alterations (CNAs) were identified using CNVkit (version 0.9.10, <https://cnvkit.readthedocs.io/en/stable/>) with default parameters. For insertion and deletion (InDel) variants, Pindel (version v0.2.5b8, <http://gmt.genome.wustl.edu/packages/pindel/>) and FLT3_ITD_ext (version 1.1, <https://github.com/ht50/FLT3_ITD_ext>) were employed on exons 13-15 to detect FLT3 internal tandem duplication (ITD) alleles.

**Variant filtering**

Variants were filtered using public databases to exclude germline mutations with a frequency greater than 0.5%. The following databases were utilized:

dbSNP (version snp147, <http://www.ncbi.nlm.nih.gov/snp>)

1000 Genomes (version 20130502, <http://browser.1000genomes.org>)

ExAC (version 0.3.1, http://exac.broadinstitute.org)

ESP6500SI-V2 (version v.0.0.25, <http://evs.gs.washington.edu/EVS/>)

Subsequently, an in-house blacklist was applied to remove false-positive variants, and a custom hotspot library was used for filtering blood cancer-related mutations.

**NGS detection panel**

The detailed list of mutation genes detected by NGS is as follow:

For patients from 2019.1.2 to 2019.11.4:

ABL1,ANKRD26,ARID1A,ASXL1,ASXL2,ATG2B,ATM,B2M,BCL2,BCL6,BCOR,BCORL1,BIRC3,BRAF,BRINP3,BTK,CALR,CARD11,CASP8,CBL,CCND1,CCND2,CCND3,CCR4,CD28,CD58,CD79B,CDC25C,CDKN1B,CDKN2A,CEBPA,CNOT3,CREBBP,CRLF2,CSF3R,CSNK1A1,CUX1,CXCR4,DDX3X,DDX41,DIS3,DNM2,DNMT3A,DNMT3B,EED,EGR1,EP300,ETNK1,ETV6,EZH2,FAM46C,FAT1,FBXW7,FGFR3,FLT3,GATA1,GATA2,GATA3,GNA13,ID3,IDH1,IDH2,IKZF1,IL7R,IRF4,JAK1,JAK2,JAK3,KDM6A,KIT,KLF2,KMT2A,KMT2D,KRAS,MAP2K1,MAPK1,MAX,MED12,MEF2B,MPL,MYC,MYD88,NF1,NOTCH1,NOTCH2,NOTCH3,NOTCH4,NPM1,NRAS,NT5C2,PAX5,PDGFRB,PHF6,PIGA,PLCG1,PLCG2,PPM1D,PRDM1,PRKCB,PRPS1,PTEN,PTPN11,RAD21,RBBP6,RELN,RHOA,RPL10,RUNX1,SETBP1,SETD2,SF1,SF3B1,SH2B3,SMC1A,SMC3,SPEN,SRP72,SRSF2,STAG2,STAT3,STAT5B,SUZ12,TAL1,TCF3,TERT,TET2,TNFAIP3,TNFRSF14,TP53,TPMT,TRAF3,U2AF1,USP7,WHSC1,WT1,XPO1,ZBTB7A,ZMYM3,ZRSR2

For patients from 2019.11.4 to 2020.2.17:

ABL1,ANKRD26,ARID1A,ASXL1,ASXL2,ATG2B,ATM,B2M,BCL2,BCL6,BCOR,BCORL1,BIRC3,BRAF,BRINP3,BTK,CALR,CARD11,CASP8,CBL,CCND1,CCND2,CCND3,CCR4,CD28,CD58,CD79B,CDC25C,CDKN1B,CDKN2A,CEBPA,CNOT3,CREBBP,CRLF2,CSF3R,CSNK1A1,CUX1,CXCR4,DDX3X,DDX41,DIS3,DNM2,DNMT3A,DNMT3B,EED,EGR1,EP300,ETNK1,ETV6,EZH2,FAM46C,FAT1,FBXW7,FGFR3,FLT3,GATA1,GATA2,GATA3,GNA13,ID3,IDH1,IDH2,IKZF1,IL7R,IRF4,JAK1,JAK2,JAK3,KDM6A,KIT,KLF2,KMT2A,KMT2D,KRAS,MAP2K1,MAPK1,MAX,MED12,MEF2B,MPL,MYC,MYD88,NF1,NOTCH1,NOTCH2,NOTCH3,NOTCH4,NPM1,NRAS,NT5C2,PAX5,PDGFRB,PHF6,PIGA,PLCG1,PLCG2,PPM1D,PRDM1,PRKCB,PRPF8,PRPS1,PTEN,PTPN11,RAD21,RBBP6,RELN,RHOA,RPL10,RUNX1,SETBP1,SETD2,SF1,SF3B1,SH2B3,SMC1A,SMC3,SPEN,SRP72,SRSF2,STAG2,STAT3,STAT5B,SUZ12,TAL1,TCF3,TERT,TET2,TNFAIP3,TNFRSF14,TP53,TPMT,TRAF3,U2AF1,USP7,WHSC1,WT1,XPO1,ZBTB7A,ZMYM3,ZNF384,ZRSR2

For patients from 2020.2.17 to 2021.4.6:

ABCB1,ABL1,ANKRD26,APC,ARID1A,ARID1B,ARID2,ASXL1,ATG2B,ATM,ATRX,B2M,BCL10,BCL2,BCL6,BCOR,BCORL1,BIRC3,BLM,BPGM,BRAF,BRCA1,BRCA2,BRIP1,BTG1,BTK,CALR,CARD11,CBL,CBLB,CBLC,CCND1,CCND3,CD28,CD58,CD79A,CD79B,CDKN1A,CDKN2A,CDKN2B,CEBPA,CHD8,CIITA,CREBBP,CRLF2,CSF1R,CSF3R,CTCF,CUX1,CXCR4,DDX41,DIS3,DKC1,DNM2,DNMT3A,EED,EGFR,SYK,EGLN1,ELANE,EP300,EPHA7,EPOR,ETV6,EZH2,FAM46C,FAS,FAT1,FBXO11,FBXW7,FLT3,FOXO1,GATA1,GATA2,GFI1,GNA13,GNAI2,GNAS,GNB1,GSKIP,HAX1,HRAS,ID3,IDH1,IDH2,IKZF1,IKZF2,IKZF3,IL7R,IRF4,IRF8,ITPKB,JAK1,JAK2,JAK3,KDM6A,KIT,KMT2A,KMT2B,KMT2C,KMT2D,KRAS,KRT20,LMO2,LYN,MAP2K1,MCL1,MEF2B,MFHAS1,MPL,MTOR,MYC,MYD88,NF1,NOTCH1,NOTCH2,NPM1,NRAS,NT5C2,PALB2,PAX5,PDGFRA,PDGFRB,PHF6,PIGA,PIK3CA,PIK3CD,PIK3R1,PIM1,PLCG2,PPM1D,PRDM1,PRF1,PRKDC,PRPF8,PTEN,PTPN11,RAD21,RELN,RHOA,RUNX1,SBDS,SETBP1,SETD2,SETDB1,SF3B1,SGK1,SH2B3,SMC1A,SMC3,SOCS1,SRP72,SRSF2,STAG2,STAT3,STAT5B,STAT6,SUZ12,TAL1,TCF3,TERC,TERT,TET2,TNFAIP3,TNFRSF14,TP53,TPMT,TRAF3,U2AF1,VHL,WHSC1,WT1,XPO1,ZAP70,ZRSR2

For patients from 2021.4.6 to 2023.5.6:

ABCB1,ABL1,ANKRD26,APC,ARID1A,ARID1B,ARID2,ARID5B,ASXL1,ASXL2,ATG2B,ATM,ATRX,B2M,BACH2,BCL10,BCL2,BCL6,BCL7A,BCOR,BCORL1,BIRC3,BLM,BPGM,BRAF,BRCA1,BRCA2,BRIP1,BTG1,BTG2,BTK,CALR,CARD11,CBL,CBLB,CBLC,CCND1,CCND3,CCR4,CD28,CD58,CD79A,CD79B,CDC25C,CDKN1A,CDKN1B,CDKN2A,CDKN2B,CDKN2C,CEBPA,CHD2,CHD8,CIITA,CNOT3,CREBBP,CRLF2,CSF1R,CSF3R,CSMD1,CSNK1A1,CTCF,CUX1,CXCR4,CYLD,DDX3X,DDX41,DIS3,DKC1,DNM2,DNMT3A,DNMT3B,DTX1,DUSP2,EBF1,EED,EGFR,EGLN1,EGR1,ELANE,EP300,EPHA7,EPOR,ETNK1,ETV6,EZH2,FAM46C,FAS,FAT1,FAT4,FBXO11,FBXW7,FGFR3,FLT3,FOXO1,FYN,GAB2,GATA1,GATA2,GATA3,GFI1,GNA13,GNAI2,GNAS,GNB1,GSKIP,H1-2,H1-3,H1-4,H1-5,HAX1,HLA-A,HLA-C,HLA-DMB,HNRNPK,HRAS,HUWE1,HVCN1,ID3,IDH1,IDH2,IGLL5,IKZF1,IKZF2,IKZF3,IL7R,IRF2BP2,IRF4,IRF8,ITPKB,JAK1,JAK2,JAK3,JUNB,KDM6A,KIT,KLF2,KLHL6,KMT2A,KMT2B,KMT2C,KMT2D,KRAS,KRT20,LCOR,LMO2,LYN,MAP2K1,MAPK1,MAX,MCL1,MED12,MEF2B,MFHAS1,MPL,MTOR,MYC,MYCN,MYD88,MYOM2,NF1,NOTCH1,NOTCH2,NOTCH3,NOTCH4,NPM1,NRAS,NT5C2,P2RY8,PALB2,PAX5,PDGFRA,PDGFRB,PHF6,PIGA,PIK3CA,PIK3CD,PIK3R1,PIM1,PIM2,PLCG1,PLCG2,POT1,PPM1D,PRDM1,PRF1,PRKCB,PRKD2,PRKDC,PRPF8,PRPS1,PSMB5,PTEN,PTPN1,PTPN11,PTPRD,RAD21,RASA2,RB1,RBBP6,RELN,RHOA,RPL10,RRAGC,RUNX1,SAMHD1,SBDS,SETBP1,SETD1B,SETD2,SETDB1,SF1,SF3B1,SGK1,SH2B3,SH2D1A,SMARCA4,SMC1A,SMC3,SMO,SOCS1,SP140,SPEN,SRP72,SRSF2,STAG2,STAT3,STAT5B,STAT6,SUFU,SUZ12,SYK,TAL1,TBL1XR1,TCF3,TERC,TERT,TET1,TET2,TMEM30A,TMSB4X,TNFAIP3,TNFRSF14,TOX,TP53,TPMT,TRAF3,U2AF1,U2AF2,UBA1,UBE2A,UBR5,USP7,VAV1,VHL,WHSC1,WT1,XBP1,XPO1,ZAP70,ZBTB7A,ZFP36L1,ZMYM3,ZNF292,ZNF318,ZNF516,ZRSR2

For patients from 2023.5.6 to now:

ABCB1,ABL1,ABL2,ACTB,ACTG1,ALDH18A1,ANKRD26,APC,ARID1A,ARID1B,ARID2,ARID5B,ASXL1,ASXL2,ATG2B,ATM,ATP13A4,ATR,ATRX,B2M,BACH2,BCL10,BCL11A,BCL2,BCL2L1,BCL6,BCL7A,BCOR,BCORL1,BIRC3,BLM,BPGM,BRAF,BRCA1,BRCA2,BRCC3,BRIP1,BTG1,BTG2,BTK,CALR,CARD11,CBL,CBLB,CBLC,CCND1,CCND2,CCND3,CCR4,CD28,CD58,CD70,CD79A,CD79B,CD83,CDC25C,CDKN1A,CDKN1B,CDKN2A,CDKN2B,CDKN2C,CEBPA,CHD2,CHD8,CHEK2,CHST2,CIITA,CLTC,CNOT3,CNPY3,CRBN,CREBBP,CRLF2,CSF1R,CSF3R,CSMD1,CSNK1A1,CTCF,CUX1,CXCR4,CYLD,DAZAP1,DDX3X,DDX41,DIS3,DKC1,DNAH11,DNM2,DNMT3A,DNMT3B,DOCK8,DTX1,DUSP2,DUSP22,EBF1,EDRF1,EED,EGFR,EGLN1,EGR1(EIF4A2,ELANE,EP300,EPB41,EPHA7,EPOR,ETNK1,ETS1,ETV6,EZH2,FANCA,FANCC,FANCG,FAS,FAT1,FAT3,FAT4,FBXO11,FBXW7,FGFR1,FGFR3,FLT3,FOXC1,FOXO1,FYN,GAB2,GATA1,GATA2,GATA3,GFI1,GNA13,GNAI2,GNAS,GNB1,GRHPR,GSKIP,H1-2,H1-3,H1-4,H1-5,H2BC12(HIST1H2BK),HAX1,HLA-A,HLA-B,HLA-C,HLA-DMB,HLA-DPB1,HLA-DQB1,HLA-DRB1,HNRNPK,HRAS,HUWE1,HVCN1,ID3,IDH1,IDH2,IGLL5,IKBKB,IKZF1,IKZF2,IKZF3,IL10RA,IL16,IL4R,IL7R,ING1,INO80,IRF2BP2,IRF4,IRF8,ITPKB,JAK1,JAK2,JAK3,JUNB,KDM6A,KIT,KLF2,KLHL14,KLHL21,KLHL42,KLHL6,KMT2A,KMT2B,KMT2C,KMT2D,KRAS,KRT20,LCOR(C10orf12),LMO2,LRRK2,LTB,LYN,MAF,MAFB,MAP2K1,MAP3K1,MAP3K14,MAP3K7,MAPK1,MAX,MBD4,MCL1,MECOM,MED12,MED16,MEF2B,MFHAS1,MGA,MPEG1,MPL,MSH2,MTOR,MYC,MYCN,MYD88,MYOM2,NF1,NFE2,NFKBIA,NFKBIE,NFKBIZ,NOL9,NOTCH1,NOTCH2,NOTCH3,NOTCH4,NPM1,NRAS,NSD2(WHSC1),NT5C2,OSBPL10,P2RY8,PABPC1,PALB2,PAX5,PDGFRA,PDGFRB,PDS5B,PHF6,PIGA,PIK3CA,PIK3CD,PIK3R1,PIM1,PIM2,PLCG1,PLCG2,POT1,PPM1D,PPP1R9B,PRDM1,PRF1,PRKCB,PRKD2,PRKDC,PRPF40B,PRPF8,PRPS1,PRRC2C,PSMB5,PTEN,PTPN1,PTPN11,PTPRD,RAD21,RASA2,RB1,RBBP6,REL,RELN,RFTN1,RHOA,RPL10,RRAGC,RUNX1,S1PR2,SAMD9,SAMD9L,SAMHD1,SBDS,SEC24C,SETBP1,SETD1B,SETD2,SETDB1,SF1,SF3A1,SF3B1,SGK1,SH2B3,SH2D1A,SLC1A5,SMARCA2,SMARCA4,SMC1A,SMC3,SMO,SOCS1,SP140,SPEN,SPIB,SRP72,SRSF2,STAG2,STAT1,STAT3,STAT5B,STAT6,SUFU,SUZ12,SYK,TAL1,TBL1XR1,TCF3,TENT5C(FAM46C),TERC,TERT,TET1,TET2,TET3,TMEM30A,TMSB4X,TNFAIP3,TNFRSF14,TNRC18,TOX,TP53,TP53BP1,TP63,TP73,TPMT,TRAF2,TRAF3,TRIP12,TRRAP,TYK2,U2AF1,U2AF2,UBA1,UBE2A,UBR5,USP7,VAV1,VHL,VMP1,WDR24,WEE1,WT1,XBP1,XPO1,ZAP70,ZBTB33,ZBTB7A,ZC3H12D,ZEB2,ZFHX4,ZFP36L1,ZMYM3,ZNF292,ZNF318,ZNF516,ZRSR2

**Statistical analysis**

Event-free survival (EFS) was defined as time from the date of diagnosis until failure to achieve CR after the first course of induction therapy, relapse, death, or the last follow-up, whichever came first. Relapse-free survival (RFS) was defined as time from achieving CR to first relapse, death or the date of last follow-up, whichever came first. Overall survival (OS) was defined as the interval from the date of diagnosis until death from any cause. Patient survival was evaluated via the Kaplan–Meier method, statistical significance of survival was estimated by log-rank test. R package “survival” and “survminer” were employed to conduct prognosis analysis and visualization of related results. For the comparison of continuous variables, Wilcoxon test was employed. When comparing the categorical variables, we applied chi-square test. Statistical analysis was conducted via R package “compareGroups”. Bonferroni correction was employed to adjust the p values. In the case-control matching, R package “MatchIt” was employed to match the samples. Multivariate Cox regression was employed to test the prognostic significance of DNM2 mutation in patients’ EFS and RFS. P<0.05 was considered as statistically significant.

**Figure legends**

**Figure S1. Flowchart of patient inclusion and exclusion criteria.**

**Figure S2. Mutational status of AML patients with wildtype *DNM2*.**

Mutational landscape of AML patients with wildtype *DNM2*. Each column represents a patient; each colored box indicates a specified somatic mutation. Light gray represents the wild-type cases. Bar plots indicate the mutation frequency of relevant gene. Bottom exhibited the *RUNX1::RUNX1T1* fusion gene status and CEBPA mutation status of AML patients.

**Figure S3. Prognostic significance of *DNM2* mutation in matched case control analysis**

Comparison of OS (A), RFS (C) and EFS (E) between *DNM2* mutated and wild-type AML patients. Comparison of OS (B), RFS (D) and EFS (F) censored at the time of transplantation between *DNM2* mutated and wild-type AML patients.

**Figure S4. Prognostic significance of *DNM2* mutation in AML patients of ELN Favorable subgroup.**

Comparison of OS (A), RFS (C), and EFS (E) in patients with and without DNM2 mutations in ELN Favorable subgroup. Comparison of OS (B), RFS (D), and EFS (F) censored at the time of transplantation in patients with and without DNM2 mutations in ELN Favorable subgroup.

**Figure S5. Prognostic significance of *DNM2* mutation in AML patients of ELN Intermediate subgroup.**

Comparison of OS (A), RFS (C), and EFS (E) in patients with and without DNM2 mutations in ELN Intermediate subgroup. Comparison of OS (B), RFS (D), and EFS (F) censored at the time of transplantation in patients with and without DNM2 mutations in ELN Intermediate subgroup.

**Figure S6. Prognostic significance of *DNM2* mutation in AML patients with *CEBPA* mutation.**

Comparison of OS (A), RFS (C), and EFS (E) in *CEBPA* mutated patients with and without *DNM2* mutations. Comparison of OS (B), RFS (D), and EFS (F) censored at the time of transplantation in *CEBPA* mutated patients with and without *DNM2* mutations.

**Figure S7. Prognostic significance of *DNM2* mutation in AML patients with *RUNX1::RUNX1T1* fusion gene positive.**

Comparison of OS (A), RFS (C) and EFS (E) in *RUNX1::RUNX1T1* fusion gene positive patients with and without *DNM2* mutations. Comparison of OS (B), RFS (D) and EFS (F) censored at the time of transplantation in *RUNX1-RUNX1T1* fusion gene positive patients with and without *DNM2* mutations

**Figure S8. *DNM2* prognostic significance in AML patients in multivariate analysis.**

Multivariate Cox regression to test the prognostic significance of DNM2 mutation in patients’ EFS (A) and RFS (B).

**Table legends**

**Table S1. Baseline characteristics of patients enrolled in the study**
