# Supplementary figures and images for: Dynamin2 mutations in newly diagnosed acute myeloid leukemia: clinical characteristics, and prognostic significance
Source: Exp Hematol Oncol. 2025 Mar 21;14:42. doi: 10.1186/s40164-025-00628-5 (PMC11927327; doi:10.1186/s40164-025-00628-5)

Figure S1

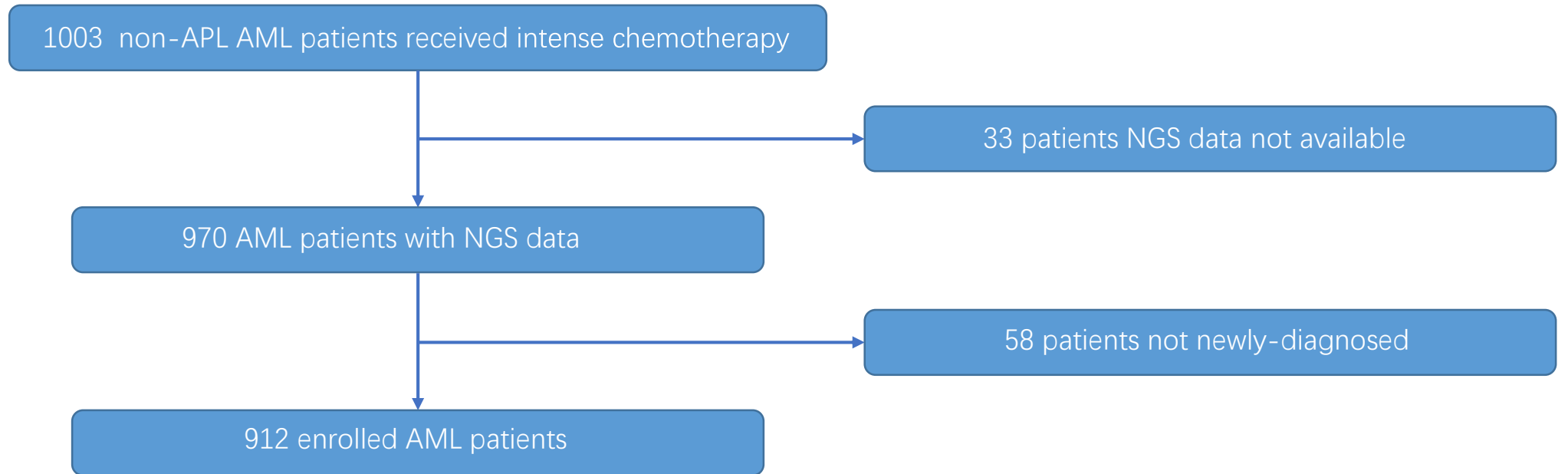

Supplement: Supplementary file 2 — Additional file 2. [file 40164_2025_628_MOESM2_ESM.pdf]

Figure S2

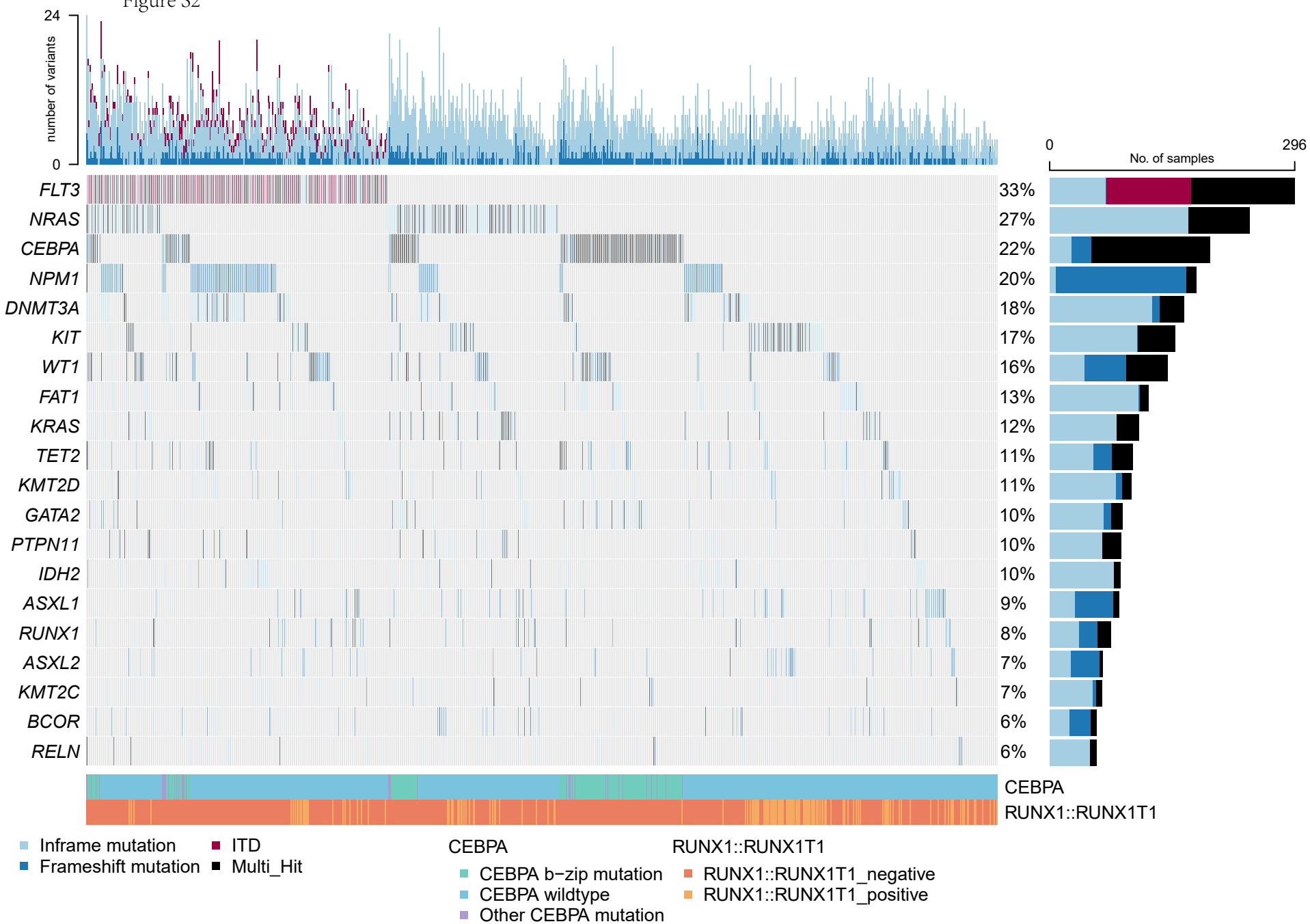

Supplement: Supplementary file 3 — Additional file 3. [file 40164_2025_628_MOESM3_ESM.pdf]

Figure S3

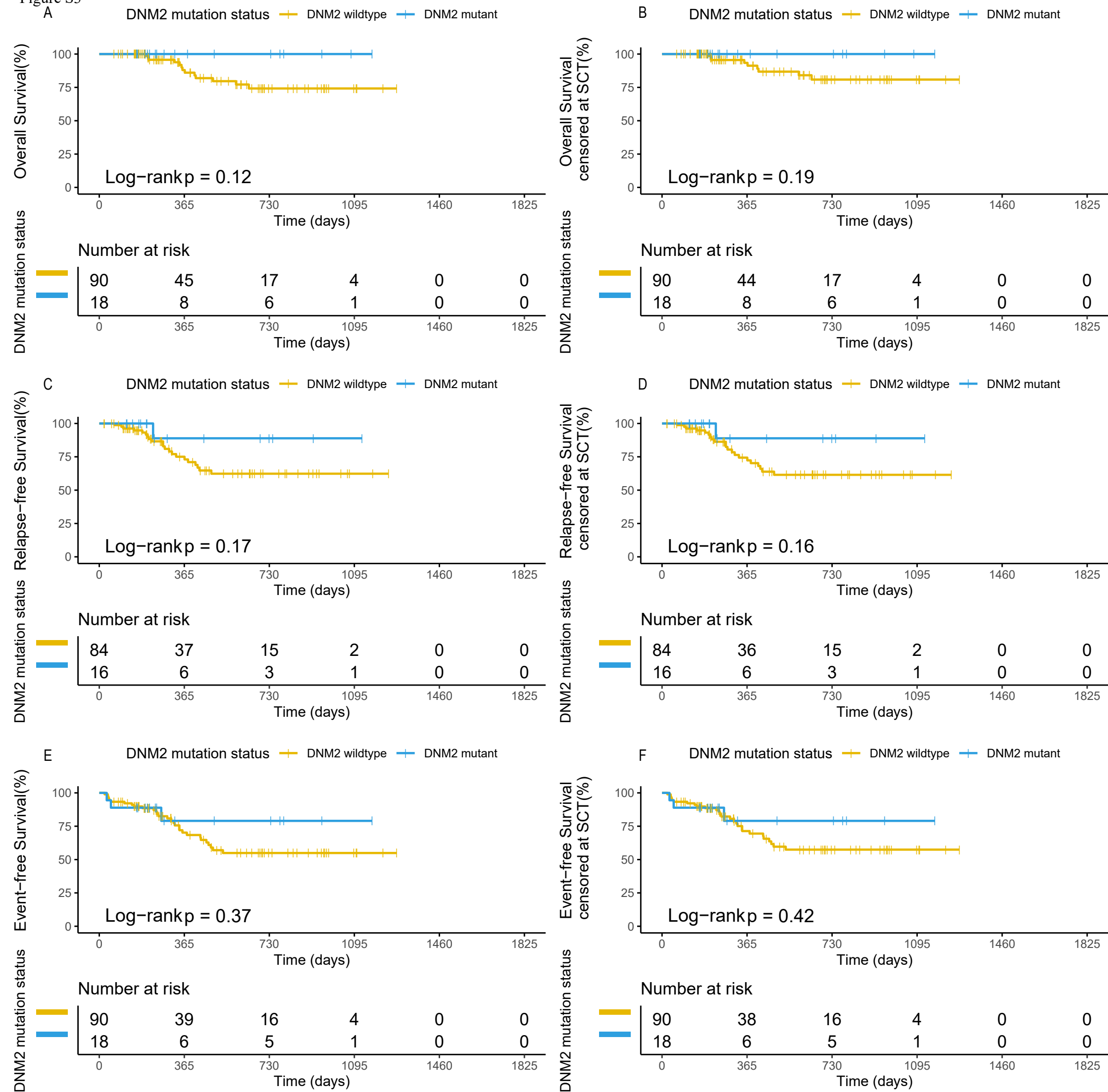

Supplement: Supplementary file 4 — Additional file 4. [file 40164_2025_628_MOESM4_ESM.pdf]

Figure S4

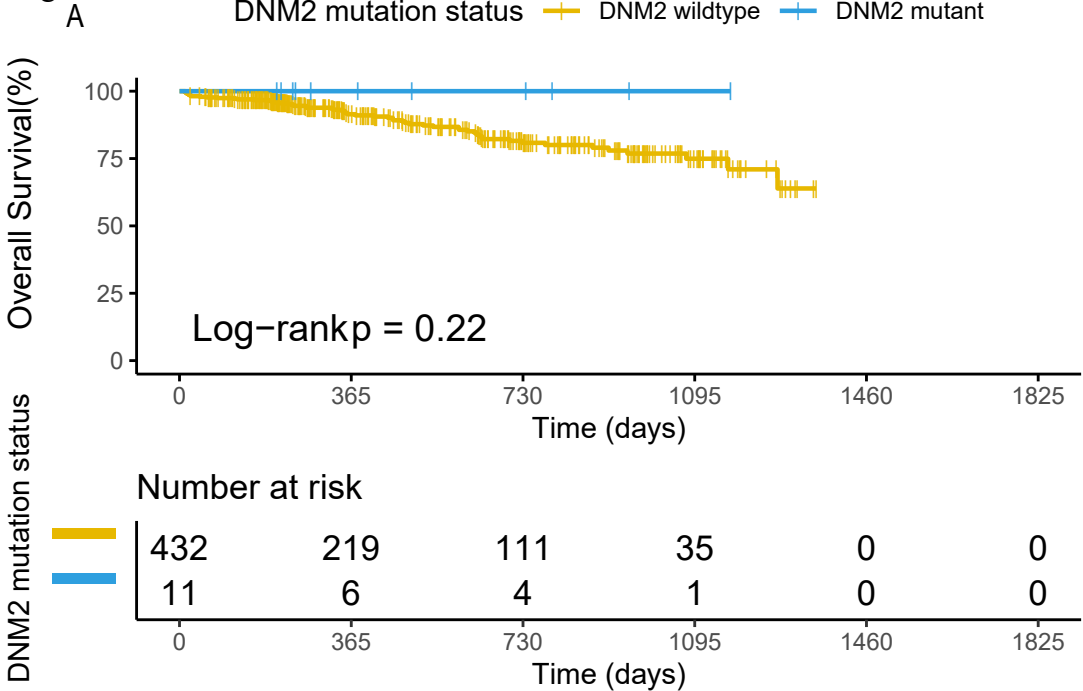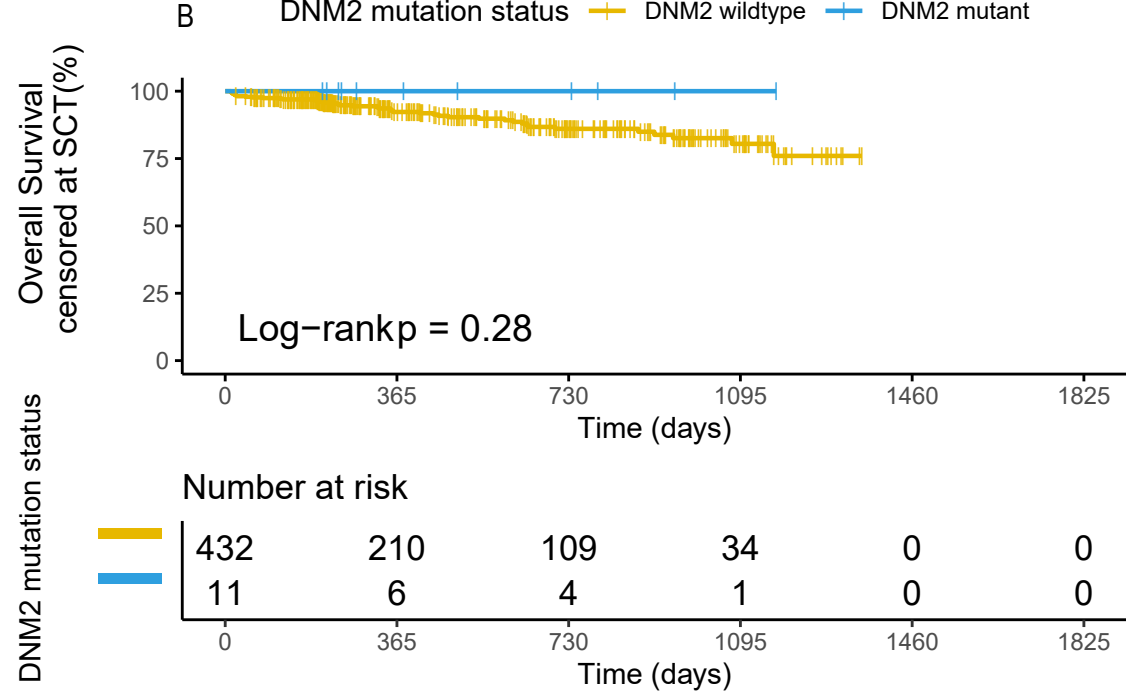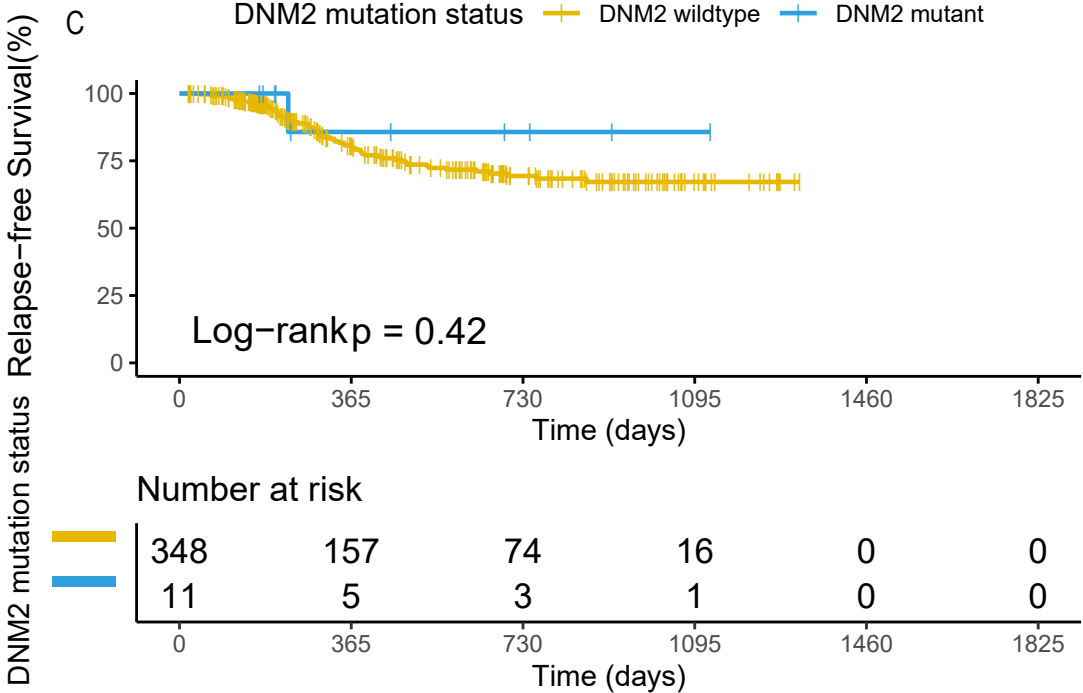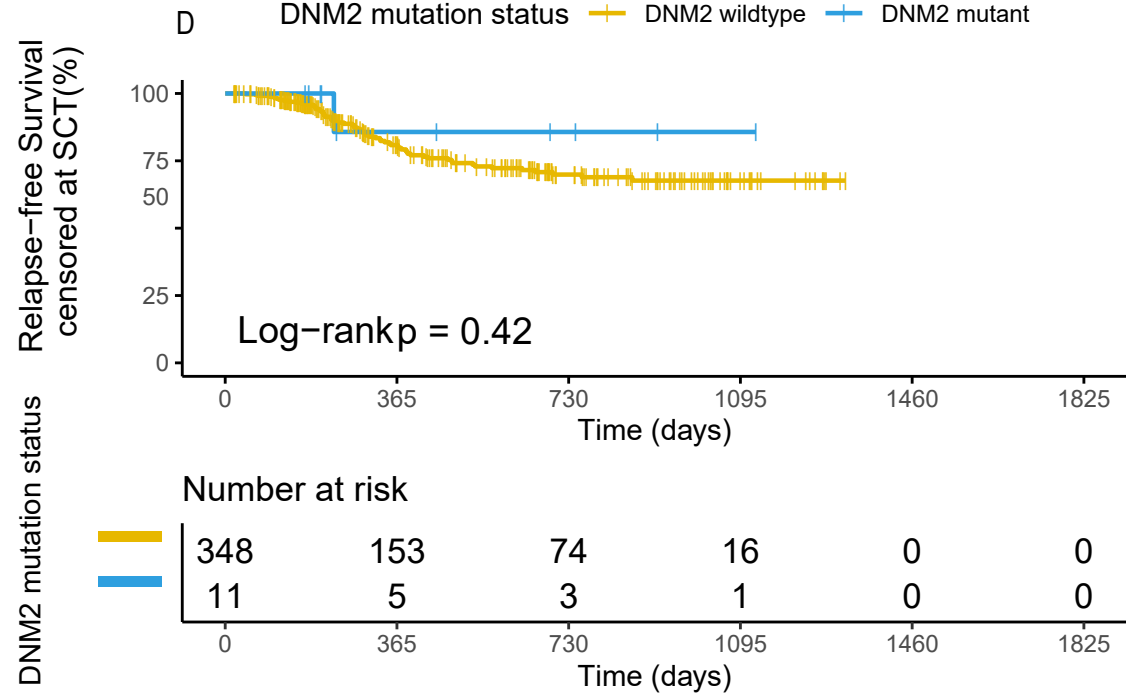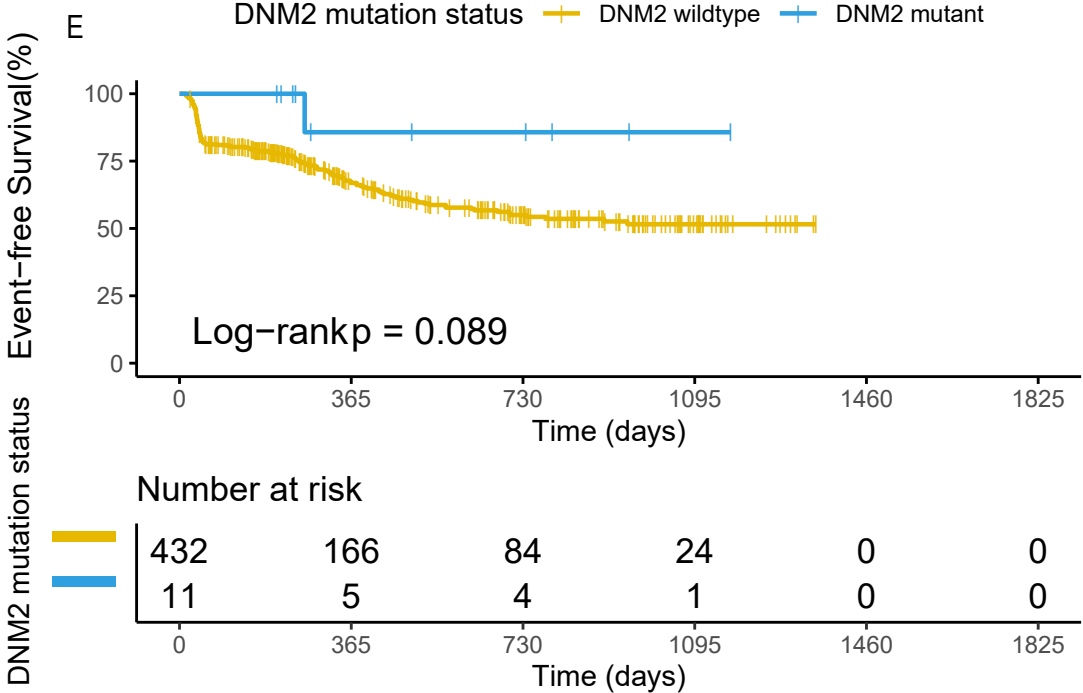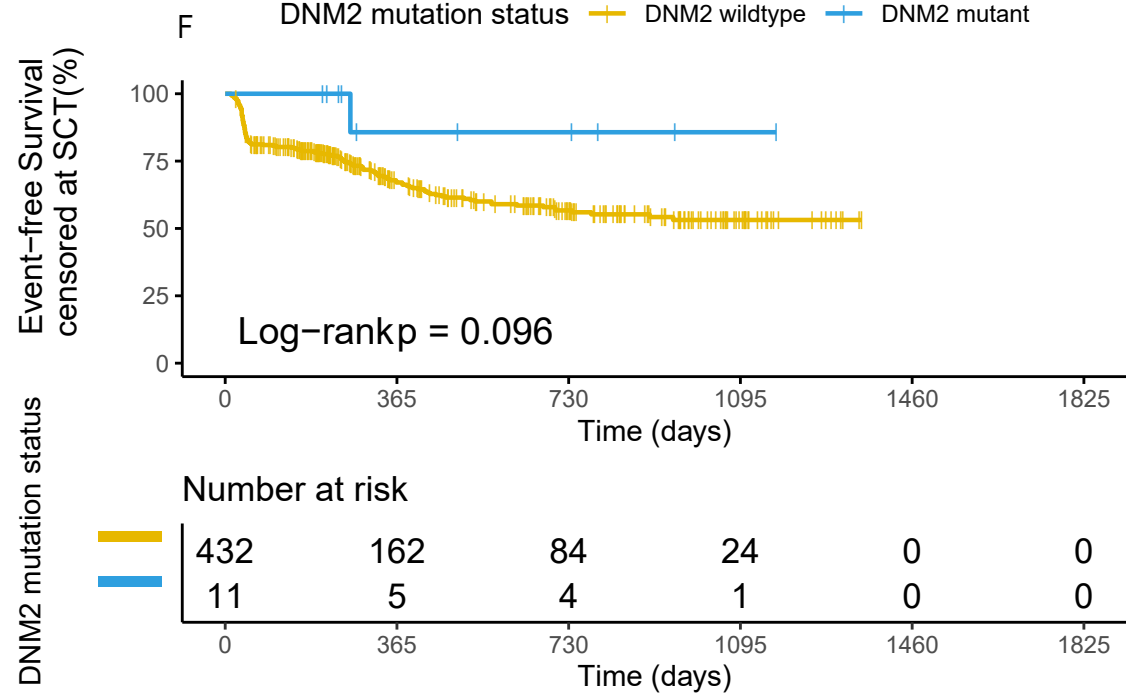

Supplement: Supplementary file 5 — Additional file 5. [file 40164_2025_628_MOESM5_ESM.pdf]

Figure S5

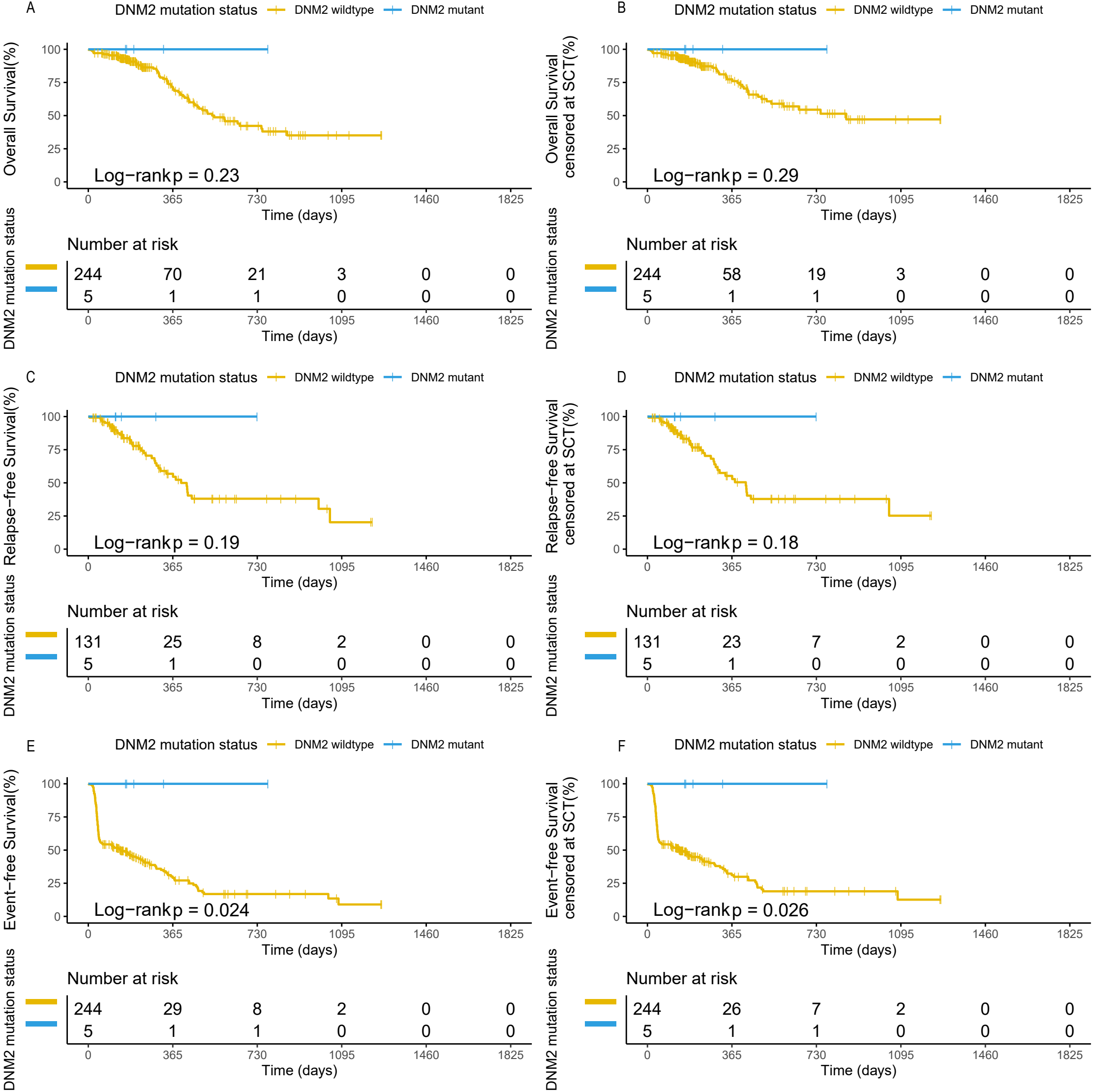

Supplement: Supplementary file 6 — Additional file 6. [file 40164_2025_628_MOESM6_ESM.pdf]

Figure S6

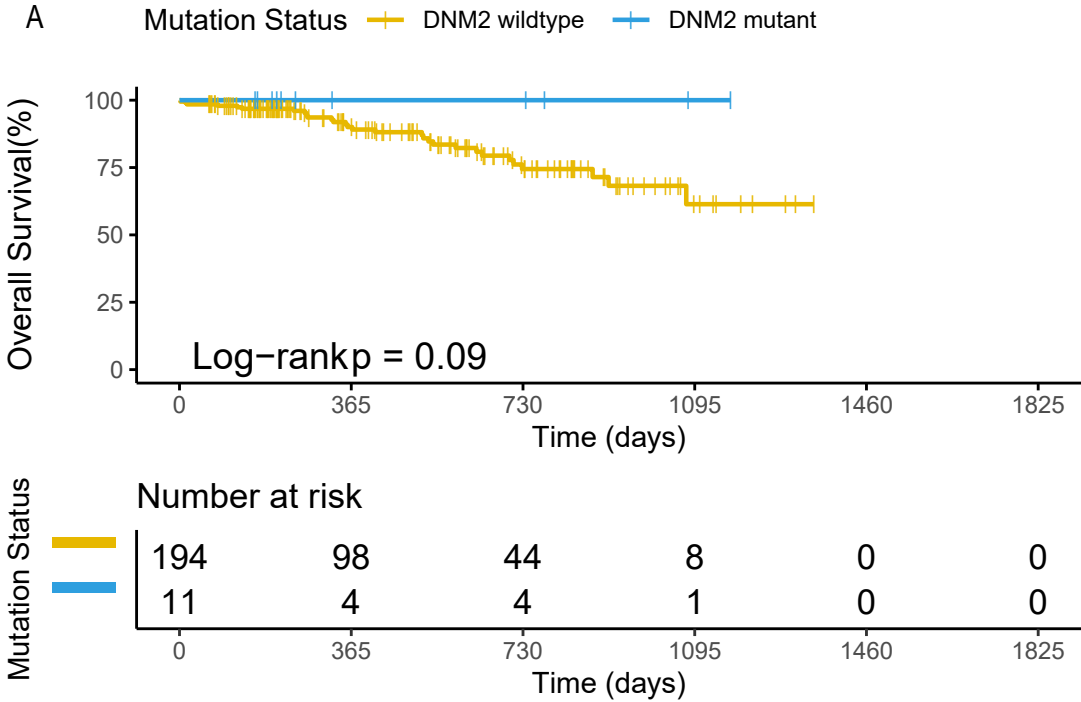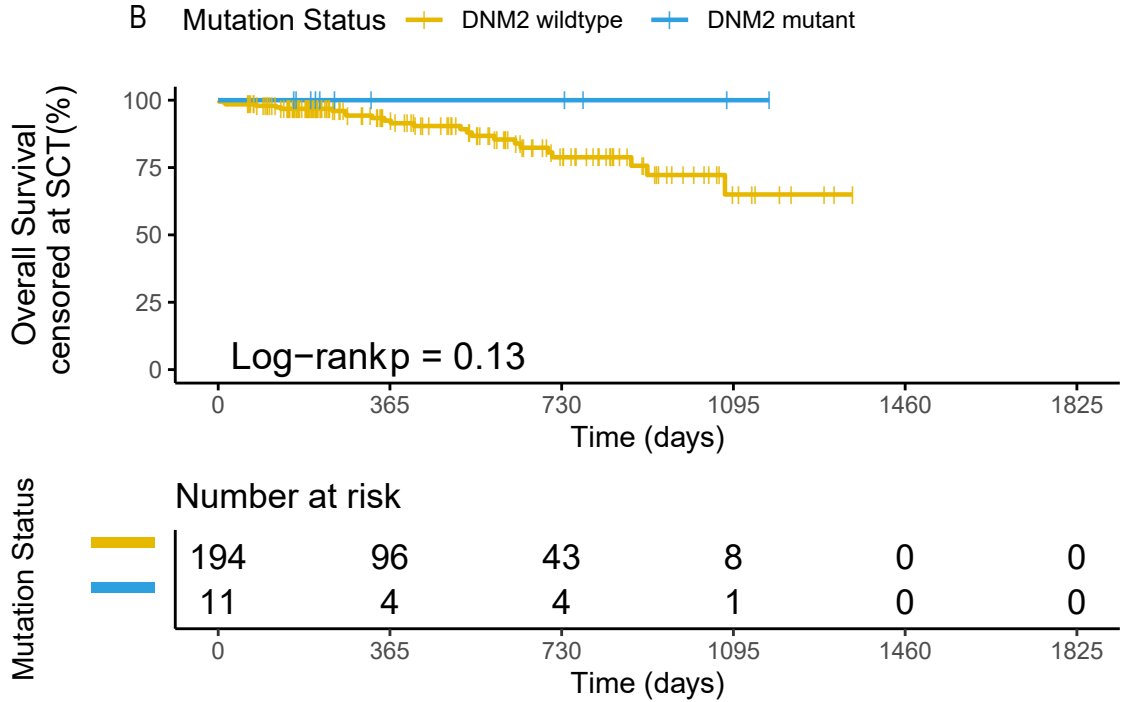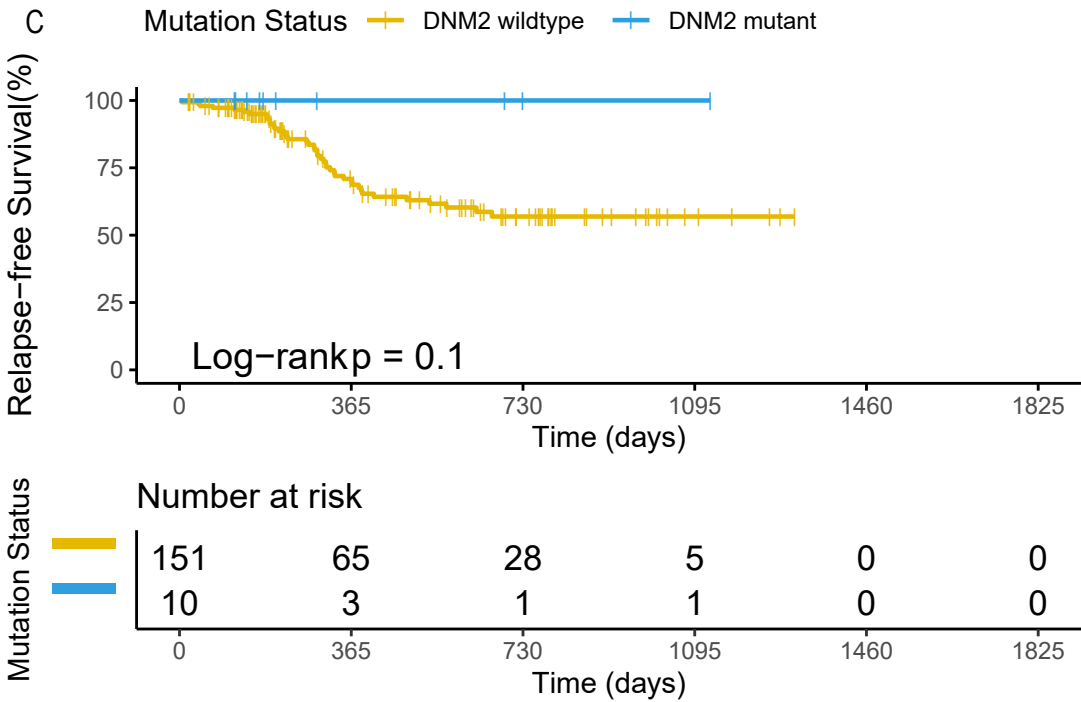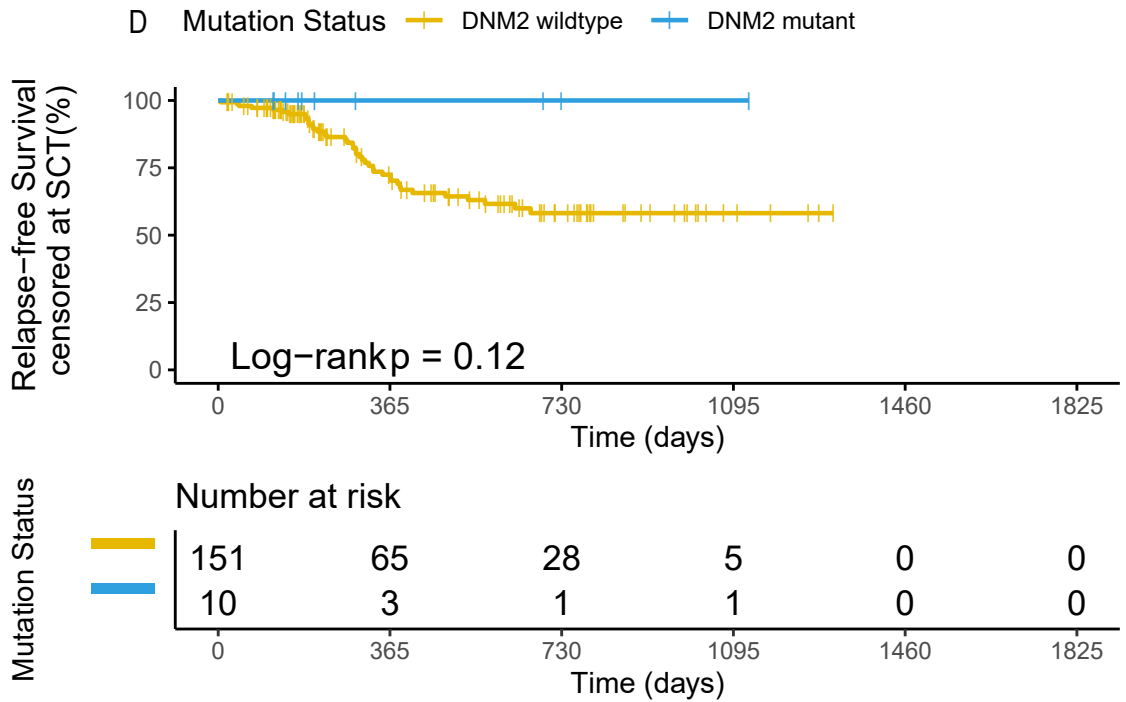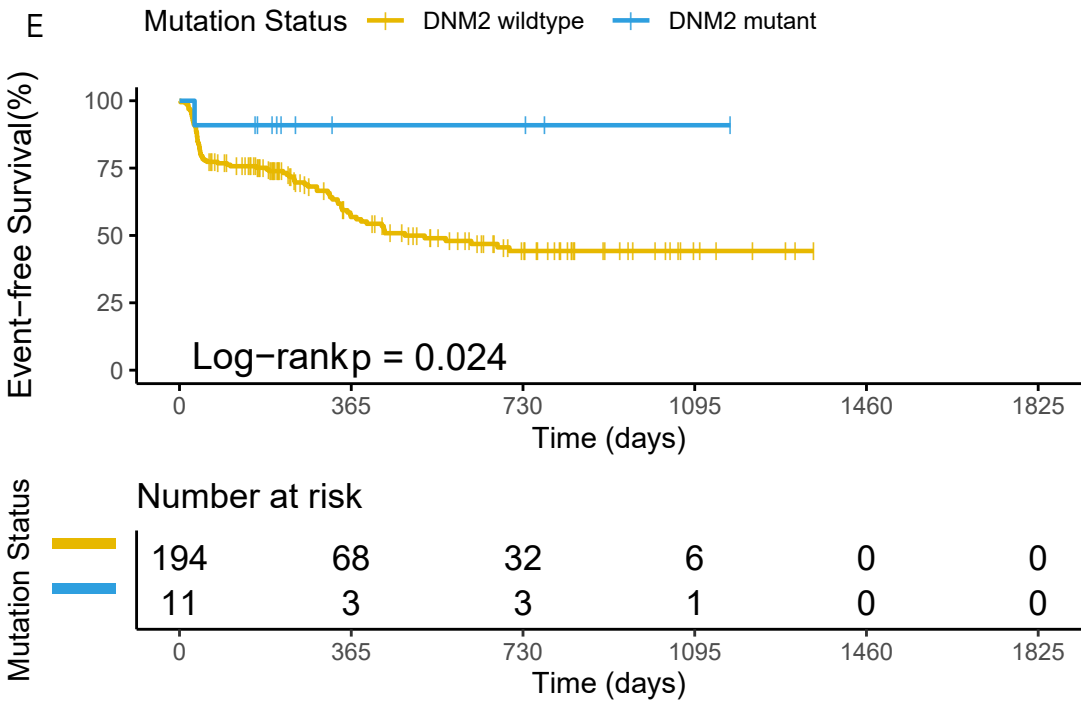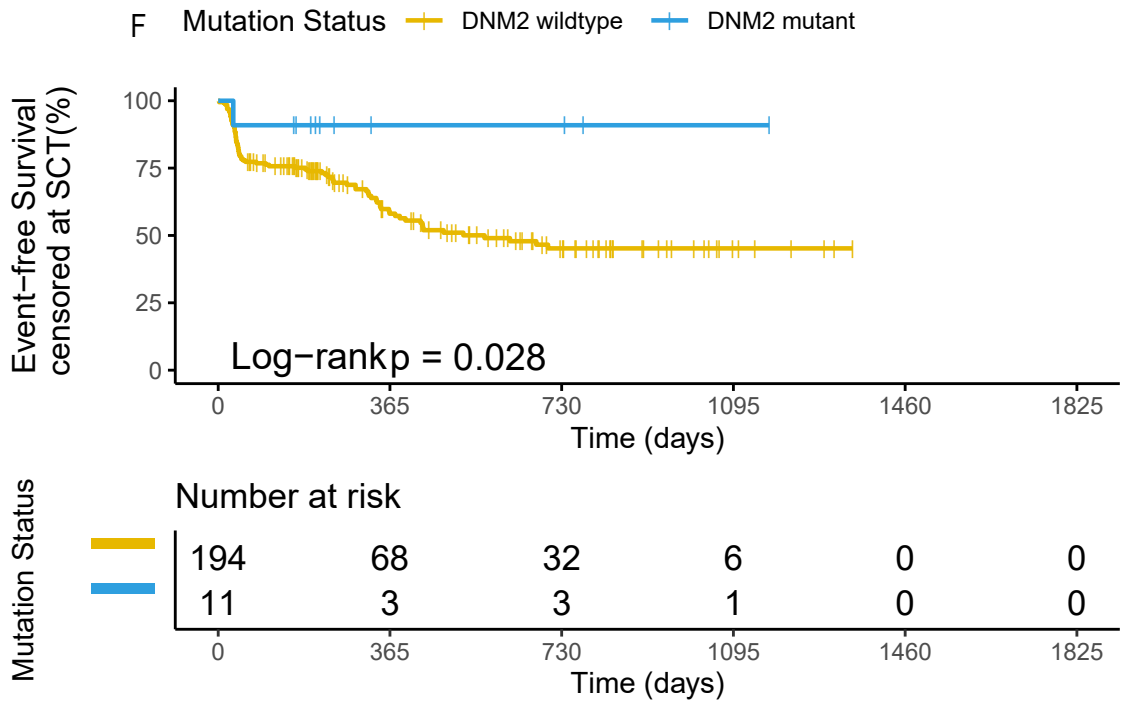

Supplement: Supplementary file 7 — Additional file 7. [file 40164_2025_628_MOESM7_ESM.pdf]

Figure S7

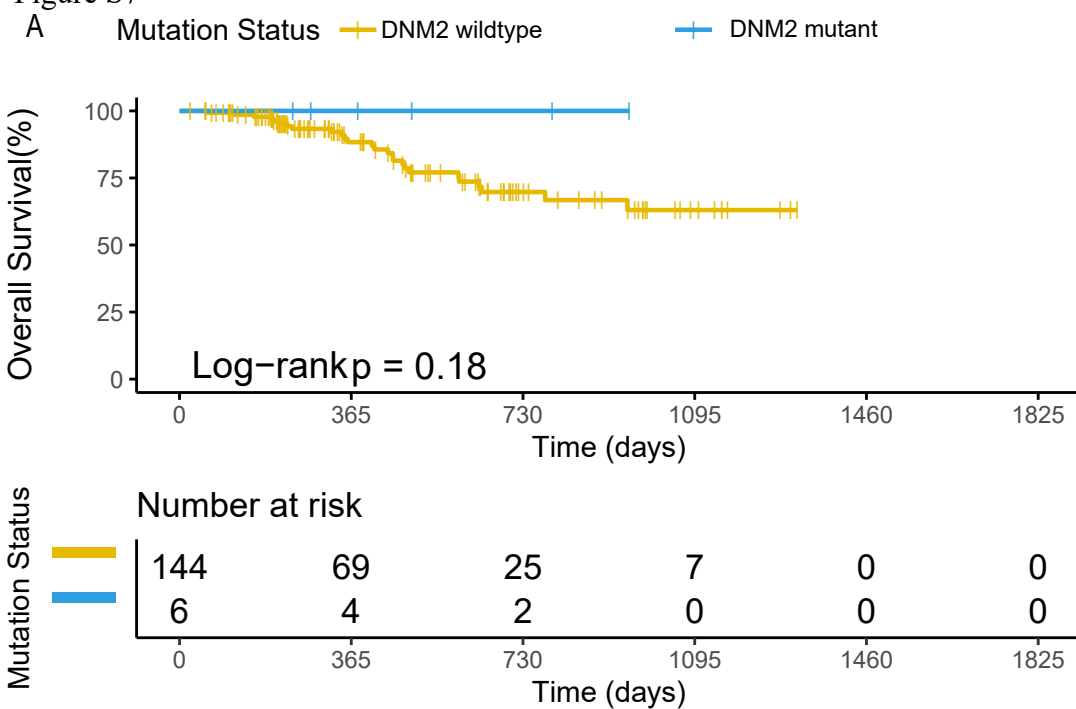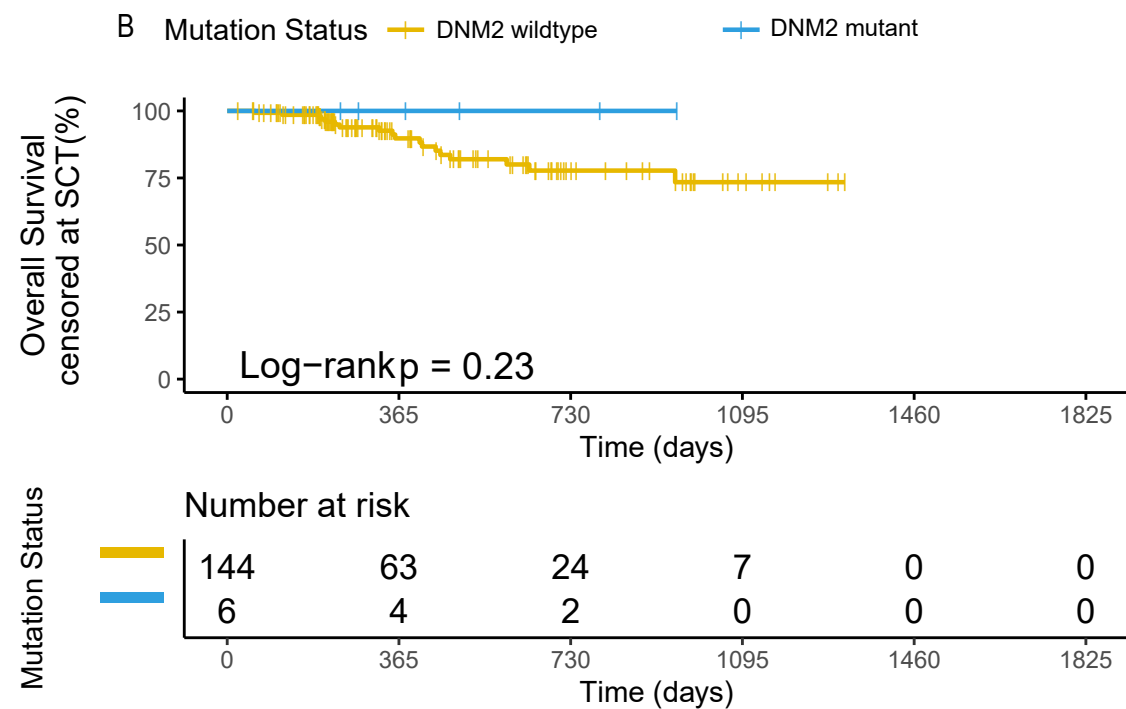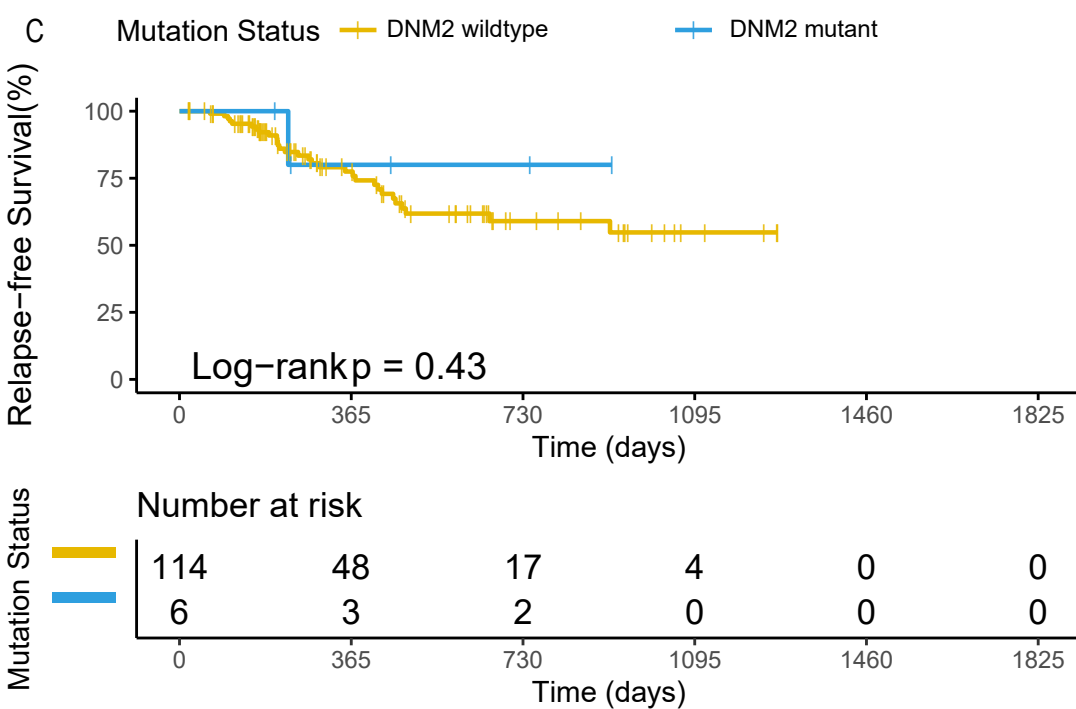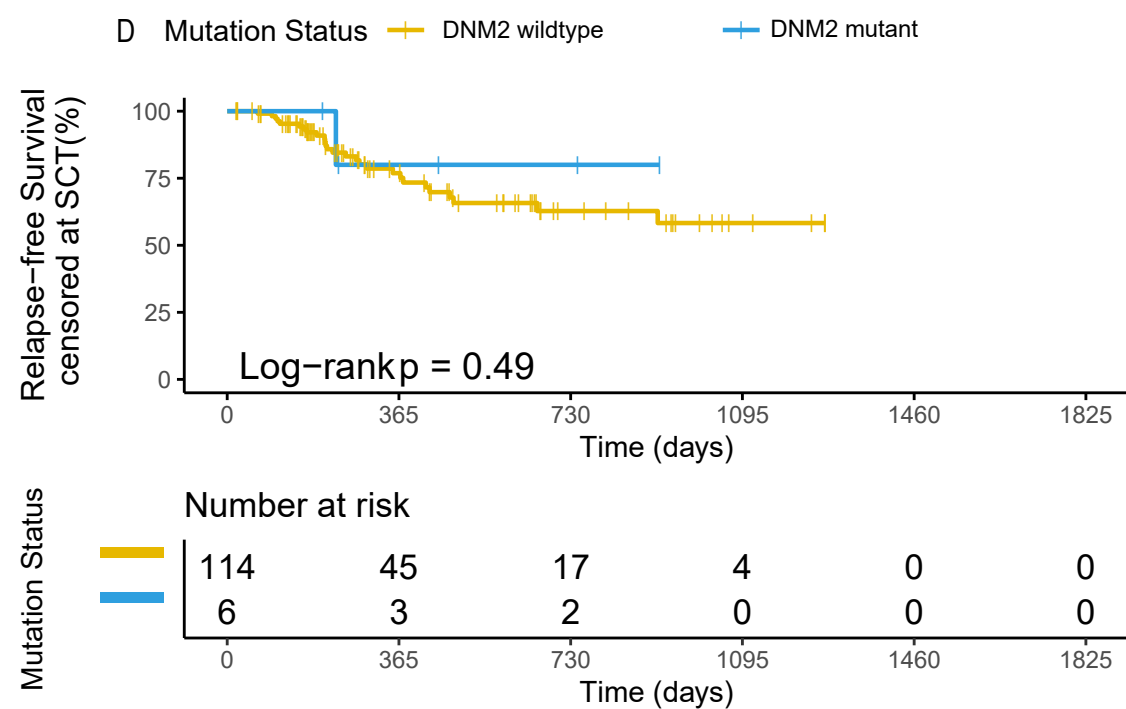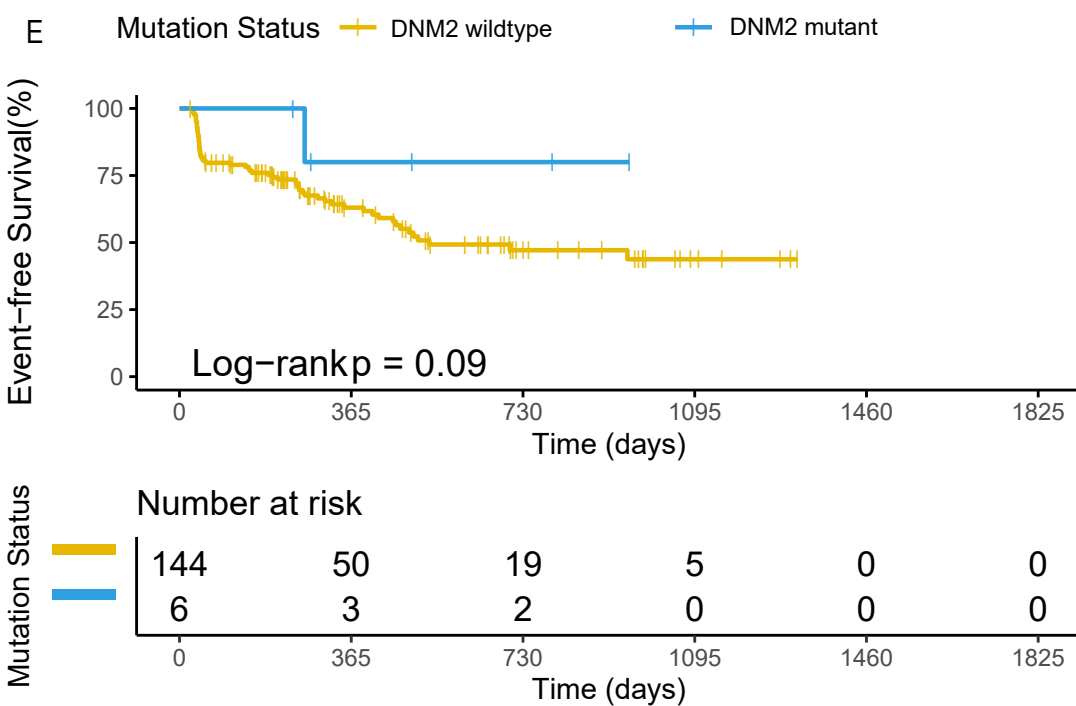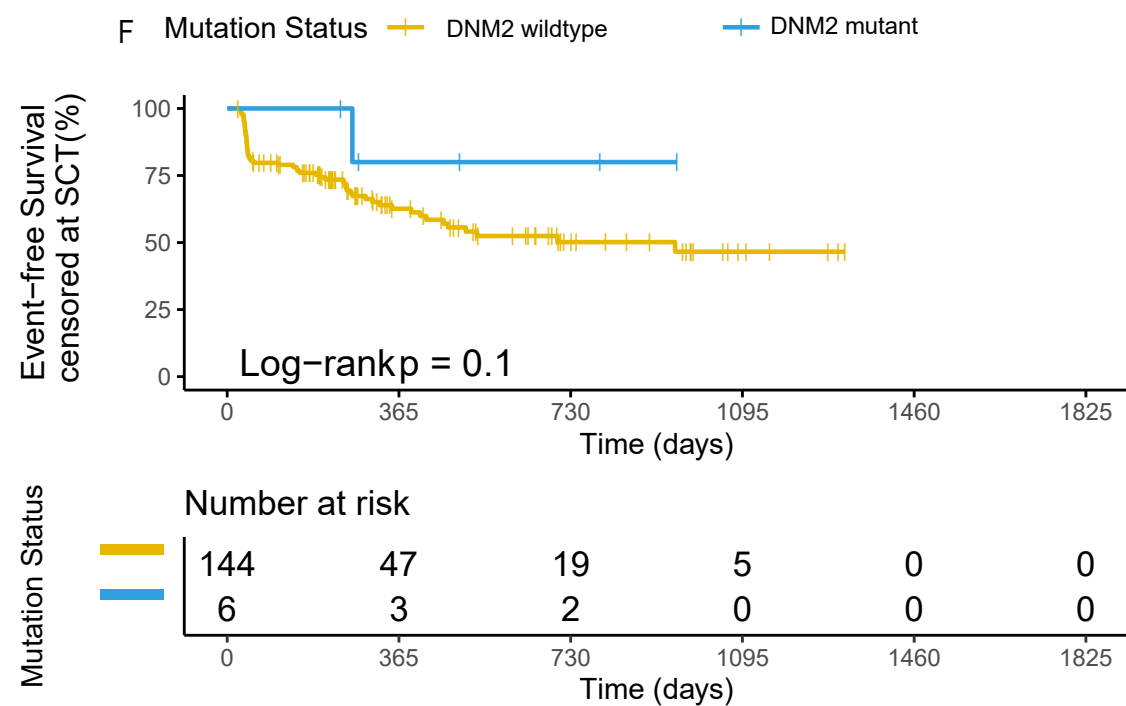

Supplement: Supplementary file 8 — Additional file 8. [file 40164_2025_628_MOESM8_ESM.pdf]

Figure S8

A

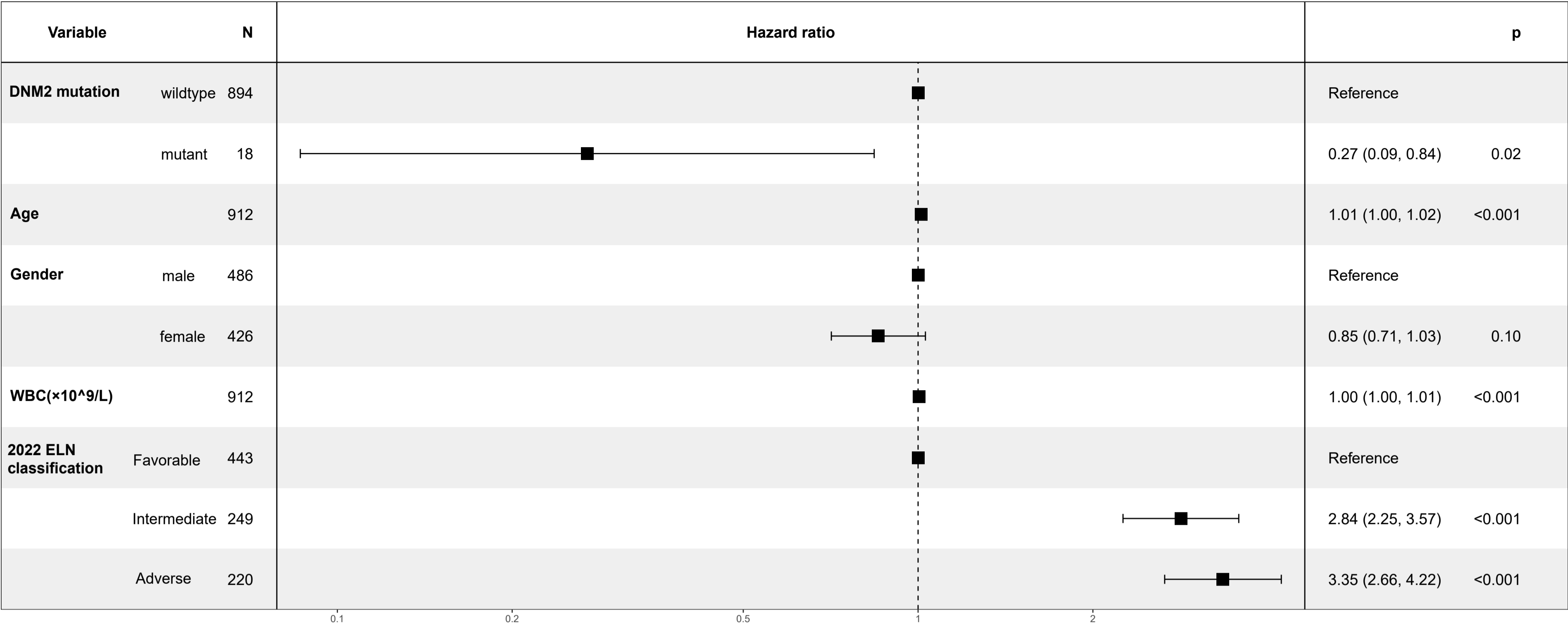

B

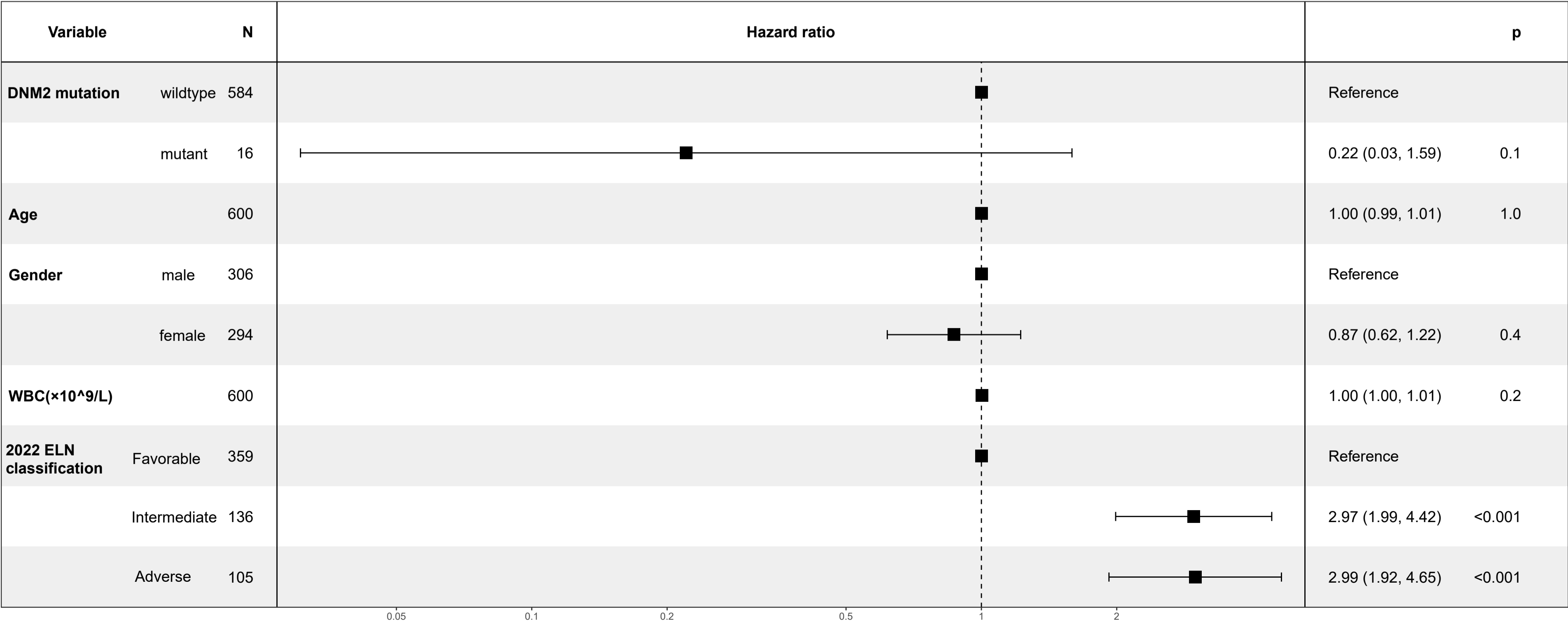

Supplement: Supplementary file 9 — Additional file 9. [file 40164_2025_628_MOESM9_ESM.pdf]
