# Supplementary material for: Dynamin2 mutations in newly diagnosed acute myeloid leukemia: clinical characteristics, and prognostic significance
Source: Exp Hematol Oncol. 2025 Mar 21;14:42. doi: 10.1186/s40164-025-00628-5 (PMC11927327; doi:10.1186/s40164-025-00628-5)
Supplement: Supplementary file 10 — Additional file 10. [file 40164_2025_628_MOESM10_ESM.docx]

|  | **DNM2 mutant** | **DNM2 wild type** | **p.adjust** |
| --- | --- | --- | --- |
|  | ***N=18*** | ***N=894*** |  |
| Age  Median (Range) | 41 (16-57) | 40 (14-68) | 0.990 |
| Gender |  |  | 0.833 |
| Female | 7 (38.9%) | 419 (46.9%) |  |
| Male | 11 (61.1%) | 475 (53.1%) |  |
| WBC(×10^9/L)  Median (Range) | 11.04 (1.19-155.97) | 15.11 (0.61-422.48) | 0.648 |
| Haemoglobin(g/L)  Median (Range) | 84.50 (49-141) | 84.8 (40-164) | 0.788 |
| Platelets(×10^9/L)  Median (Range) | 33.00 (9-123) | 41 (2-666) | 0.399 |
| CR1: |  |  | 0.274 |
| Complete remission | 16 (88.9%) | 584 (65.3%) |  |
| Not complete remission | 2 (11.1%) | 310 (34.7%) |  |
| Transplantation: |  |  | 0.833 |
| No | 13 (72.2%) | 585 (65.4%) |  |
| Yes | 5 (27.8%) | 309 (34.6%) |  |
| CEBPA: |  |  | <0.001 |
| CEBPA b−zip mutation | 7 (38.9%) | 160 (17.9%) |  |
| Other CEBPA mutation | 4 (22.2%) | 34 (3.8%) |  |
| CEBPA wildtype | 7 (38.9%) | 700 (78.3%) |  |
| RUNX1::RUNX1T1 fusion gene: | 6 (33.3%) | 144 (16.1%) | 0.326 |
| CBFB::MYH11 fusion gene | 0 (0%) | 73 (8.2%) | 0.648 |
| FLT3-ITD | 3 (16.7%) | 198 (22.1%) | 0.833 |
| NPM1 | 0 (0%) | 177 (19.8%) | 0.274 |
| TP53 | 0 (0%) | 38 (4.3%) | 0.833 |
| CSF3R | 4 (22.2%) | 47 (5.3%) | 0.063 |
| JAK3 | 4 (22.2%) | 33 (3.7%) | 0.008 |
| WT1 | 4 (22.2%) | 143 (16.0%) | 0.833 |
| ASXL2 | 3 (16.7%) | 64 (7.2%) | 0.597 |
| CSMD1 | 3 (16.7%) | 48 (5.3%) | 0.331 |
| 2022 ELN classification: |  |  | 0.648 |
| Favorable | 11 (61.1%) | 432 (48.3%) |  |
| Intermediate | 5 (27.8%) | 244 (27.3%) |  |
| Adverse | 2 (11.1%) | 218 (24.4%) |  |

|  |  |  |
| --- | --- | --- |
